# Comparison of Dupilumab and Revision Endoscopic Sinus Surgery for Recurrent Chronic Rhinosinusitis with Nasal Polyps: A Retrospective Cohort Study

**DOI:** 10.3390/jcm15135228

**Published:** 2026-07-04

**Authors:** Bartłomiej Kamiński, Dominika Ochab, Stanisław Flaga, Piotr Łacwik, Mariola Jasikowska, Cezary Pałczyński

**Affiliations:** 1 Department of Otolaryngology, Maria Skłodowska-Curie District Hospital, 26-110 Skarżysko-Kamienna, Poland; 2 Faculty of Medicine, Collegium Medicum, Jan Kochanowski University, 25-369 Kielce, Poland; 3 Clinical Division of Lung Diseases and Allergology, Holy Cross Centre for Lung Disease, 26-001 Czerwona Góra, Poland; 4 Faculty of Mechanical Engineering and Robotics, AGH University of Krakow, 30-059 Krakow, Poland

**Keywords:** chronic rhinosinusitis with nasal polyps, CRSwNP, dupilumab, biologic therapy, endoscopic sinus surgery, revision surgery

## Abstract

**Background:** Despite growing evidence supporting the efficacy of dupilumab in the treatment of chronic rhinosinusitis with nasal polyps (CRSwNP), studies directly comparing biologic therapy with revision endoscopic sinus surgery remain limited. This study aimed to compare the effectiveness of dupilumab and revision endoscopic sinus surgery (ESS) in patients with recurrent CRSwNP. **Methods:** A retrospective cohort study was conducted in 50 patients with CRSwNP who had previously undergone at least two endoscopic sinus surgeries. Twenty-three patients received dupilumab, while twenty-seven underwent revision surgery. Changes in Nasal Polyp Score (NPS), quality of life assessed using the Sino-Nasal Outcome Test-22 (SNOT-22), and olfactory function evaluated with the Sniffin’ Sticks test were analyzed. Final clinical outcomes and the proportion of patients achieving very good disease control were also assessed. **Results:** Both treatment modalities improved disease control. However, dupilumab was associated with significantly greater reductions in NPS (median [IQR]: −6.00 [−6.00 to −5.00] vs. −4.00 [−6.00 to −3.00]; *p* = 0.012), larger improvements in SNOT-22 scores (−59.39 ± 12.14 vs. −21.37 ± 20.15; *p* < 0.001), and better recovery of olfactory function (+4.65 ± 2.79 vs. +2.19 ± 2.08; *p* = 0.001) compared with revision surgery. Analysis of final outcomes also favored dupilumab, with lower NPS scores (median [IQR]: 0.00 [0.00–1.00] vs. 1.00 [0.00–2.50]; *p* = 0.023), and better olfactory function (7.65 ± 3.11 vs. 4.78 ± 2.34; *p* < 0.001). Very good symptom control (SNOT-22 < 20) was achieved by 82.6% of patients treated with dupilumab compared with 7.4% of those undergoing revision surgery. **Conclusions:** Both revision surgery and dupilumab improved disease control in patients with CRSwNP. In this cohort, dupilumab was associated with greater reductions in disease severity, superior quality-of-life outcomes, and better olfactory function than revision endoscopic sinus surgery.

## 1. Introduction

Endoscopic sinus surgery remains the primary surgical treatment for patients with chronic rhinosinusitis with nasal polyps (CRSwNP) [1]. However, despite surgical intervention and appropriate medical therapy, including intranasal corticosteroids and intermittent courses of systemic corticosteroids, CRSwNP is characterized by a high rate of recurrence. Depending on the duration of follow-up and the definition of recurrence used, polyp regrowth is observed in 20–60% of patients, with some requiring multiple revision sinus surgeries [2].

The high recurrence rate suggests that CRSwNP is not merely a localized inflammatory disorder but rather a complex immune-mediated disease predominantly driven by type 2 inflammation [3]. This process is largely orchestrated by T helper 2 (Th2) lymphocytes and group 2 innate lymphoid cells (ILC2s), resulting in the overproduction of key inflammatory mediators, particularly interleukin (IL)-5, IL-4, and IL-13, which play central roles in the initiation and maintenance of type 2 inflammatory responses [4]. IL-4 and IL-13 promote IgE-mediated immune responses through B-cell activation and contribute to tissue remodeling. IL-13, in particular, stimulates mucus hypersecretion and enhances stromal edema formation. These mediators also contribute to epithelial barrier dysfunction and the persistence of chronic inflammation [5,6,7]. Both cytokines signal through the IL-4 receptor alpha subunit (IL-4Rα), representing an important therapeutic target for modern biologic therapies.

Interleukin-5 (IL-5) plays a pivotal role in eosinophil activation, differentiation, and survival, while eosinophilic infiltration is a hallmark of type 2 inflammation in CRSwNP [8,9]. Accumulation of eosinophils within the sinonasal mucosa contributes to epithelial damage and perpetuation of chronic inflammation. These cells release numerous inflammatory mediators, including eosinophil cationic protein and galectin-10. Galectin-10 crystallization leads to the formation of Charcot–Leyden crystals (CLCs), recognized histopathological markers of eosinophilic inflammation frequently observed in nasal polyp tissue specimens [10,11].

Improved understanding of the mechanisms underlying type 2 inflammation has led to the development of biologic therapies targeting key cytokines involved in the pathogenesis of CRSwNP. In recent years, monoclonal antibodies directed against mediators of type 2 inflammation have demonstrated substantial clinical efficacy in patients with severe, uncontrolled disease. Dupilumab is a fully human monoclonal antibody targeting the IL-4Rα subunit shared by the IL-4 and IL-13 signaling pathways. By blocking signaling through both cytokines, dupilumab suppresses type 2 inflammatory activity and reduces disease burden.

Despite the growing body of evidence supporting the efficacy of dupilumab in CRSwNP, studies directly comparing biologic therapy with revision endoscopic sinus surgery remain limited [12,13,14,15,16,17,18,19,20].

The aim of this study was to compare the effectiveness of biologic therapy with dupilumab and revision endoscopic sinus surgery in patients with recurrent CRSwNP who had previously undergone at least two sinus surgeries.

## 2. Materials and Methods

A retrospective comparative study was conducted in two groups of patients who had previously undergone at least two endoscopic sinus surgeries for CRSwNP. The first group consisted of patients who underwent revision endoscopic sinus surgery, whereas the second group included patients treated with dupilumab administered at a dose of 300 mg subcutaneously every two weeks.

The decision to perform revision surgery or initiate biologic therapy was made individually according to current clinical indications. Treatment decisions were made during multidisciplinary evaluation involving rhinologists and allergists. The choice between revision ESS and dupilumab considered patient comorbidities, previous treatment history, anatomical findings, expected surgical benefit, and patient preference. The presence of type 2 inflammatory comorbidities and the feasibility of regular hospital visits required for biologic therapy were also taken into account. Treatment selection was based on clinical assessment, availability of biologic therapy, and the presence of factors that could limit the appropriateness or safety of further surgical intervention.

Inclusion criteria were age ≥ 18 years, a diagnosis of CRSwNP according to the EPOS 2020 criteria, at least two previous endoscopic sinus surgeries, treatment with intranasal corticosteroids, and at least two previous courses of systemic corticosteroids. Patients with severe uncontrolled disease and an NPS > 5 were eligible for inclusion. To ensure comparability between treatment groups, only patients fulfilling the national eligibility criteria for dupilumab reimbursement were included in the study. Accordingly, all patients had confirmed type 2 inflammation, defined by the presence of at least one of the following criteria: blood eosinophil count ≥ 150 cells/μL, total serum IgE concentration > 100 IU/mL, or >10 eosinophils per high-power field (hpf, ×400 magnification) on histopathological examination of nasal polyp tissue.

Exclusion criteria included age < 18 years, absence of evidence of type 2 inflammation, NPS ≤ 5, active parasitic infection, and severe systemic diseases precluding either surgical or biologic treatment. Patients were included between 1 September 2024 and 30 April 2026.

Clinical parameters were assessed at baseline (study inclusion) and at the last available follow-up visit performed between 12 and 18 months after treatment initiation. All patients underwent nasal endoscopy at baseline. Endoscopic assessments were performed by the treating physicians during routine clinical follow-up and were not evaluated in a blinded manner. Disease severity was assessed using the bilateral Nasal Polyp Score (NPS; range 0–8) according to EPOS 2020 recommendations. All NPS evaluations were performed by the same investigator in order to minimize interobserver variability. Quality of life was assessed using the validated Polish version of the SNOT-22 questionnaire, while olfactory function was evaluated using the 12-item Sniffin’ Sticks identification test.

Changes in NPS, SNOT-22 scores, and Sniffin’ Sticks test results between baseline and follow-up assessments were analyzed.

Revision ESS procedures were performed by a single experienced rhinologist. All patients underwent complete revision ESS, with the extent of surgery determined according to preoperative CT findings and intraoperative assessment of disease extent.

All patients received standard postoperative medical treatment according to institutional practice, including regular intranasal corticosteroids and saline nasal irrigations. Additional medical therapy, including short courses of systemic corticosteroids, was prescribed when clinically indicated.

Statistical analyses were performed using Statistica 14 software. Continuous variables are presented as mean ± standard deviation (SD), whereas categorical variables are presented as counts and percentages. Normality of data distribution was assessed using the Shapiro–Wilk test.

Comparisons between two independent groups for continuous variables with approximately normal distributions were performed using the independent-samples Student’s *t*-test. Ordinal variables and discrete count variables were compared using the Mann–Whitney U test, irrespective of data distribution. Categorical variables were analyzed using Pearson’s chi-square test or Fisher’s exact test, as appropriate.

For continuous variables, between-group differences were expressed as mean differences with corresponding 95% confidence intervals (95% CIs). For ordinal variables, data are presented as median and interquartile range (IQR), with between-group comparisons performed using the Mann–Whitney U test.

In the quality-of-life analysis, the proportion of patients achieving the minimal clinically important difference (MCID; ≥8.9-point reduction) for SNOT-22 was calculated [21]. Additionally, the proportion of patients achieving a low symptom burden, defined as SNOT-22 < 20, was assessed [22]. The proportion of patients achieving very good disease control, defined as a final NPS ≤ 1, was also analyzed. For illustrative purposes, histograms showing the distribution of changes in NPS, SNOT-22 scores, and olfactory function were generated for both treatment groups.

All statistical tests were two-sided, and a *p*-value < 0.05 was considered statistically significant.

## 3. Results

The two groups were comparable with respect to most baseline characteristics, including age, sex, body mass index (BMI), comorbidities, baseline NPS, SNOT-22 scores, and olfactory function. However, patients treated with dupilumab had undergone significantly more previous sinus surgeries than those treated with revision surgery (median [IQR]: 4.00 [2.00–5.50] vs. 2.00 [2.00–3.00]; *p* = 0.004). The mean follow-up period was comparable between the groups (14.89 ± 2.56 vs. 15.39 ± 2.44 months; *p* = 0.482) (Table 1).

**Figure 1:**
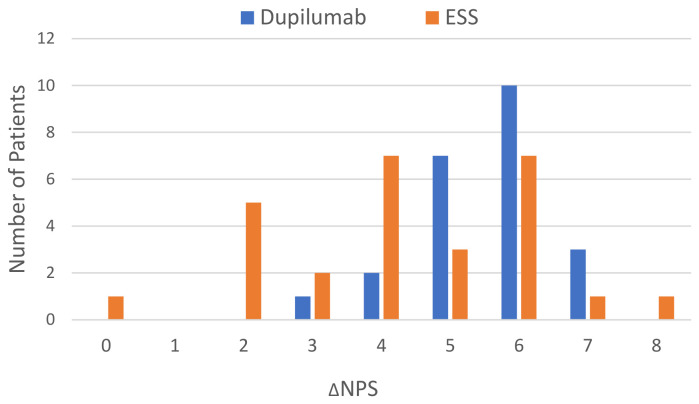
Distribution of ΔNPS values in the dupilumab and revision ESS groups.

**Figure 2:**
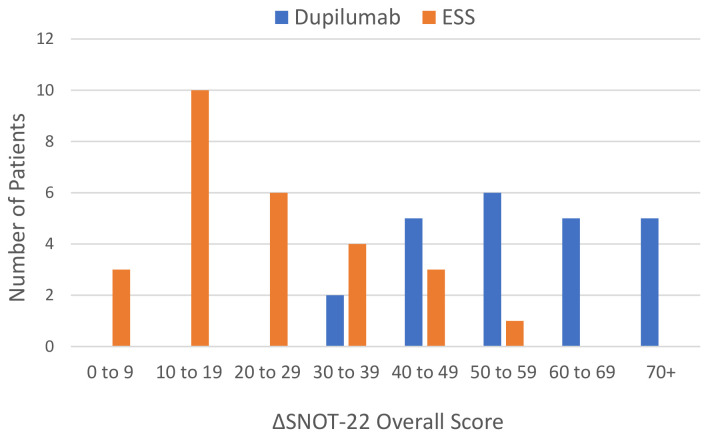
Distribution of changes in SNOT-22 scores (ΔSNOT-22) in the dupilumab and revision ESS groups.

**Figure 3:**
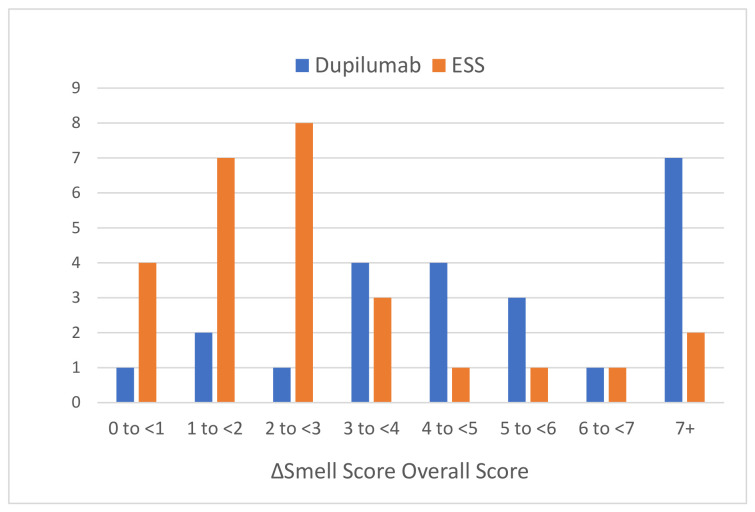
Distribution of changes in Sniffin’ Sticks scores (ΔSmell Score) in the dupilumab and revision ESS groups.

Analysis of changes from baseline confirmed the superiority of dupilumab across all three evaluated outcomes. Reduction in NPS was greater in the biologic therapy group than in the revision surgery group (median [IQR]: −6.00 [−6.00 to −5.00] vs. −4.00 [−6.00 to −3.00]; *p* = 0.012) (Figure 1). The largest difference was observed for disease-specific quality of life assessed using the SNOT-22 questionnaire, where the mean reduction was −59.39 points following dupilumab treatment compared with −21.37 points after revision sinus surgery (95% CI −46.89 to −29.15; *p* = 2.78 × 10^−11^) (Figure 2). Although both values exceeded the MCID threshold for SNOT-22, the magnitude of improvement with dupilumab was more than twofold greater. Similarly, improvement in olfactory function assessed by the Sniffin’ Sticks test was significantly greater following biologic treatment (+4.65 ± 2.79 vs. +2.19 ± 2.08; 95% CI 1.04–3.89; *p* = 0.001) (Figure 3).

Analysis of final outcome measures also demonstrated the superiority of dupilumab over revision surgery. Final NPS was significantly lower in the dupilumab group than in the revision surgery group (median [IQR]: 0.00 [0.00–1.00] vs. 1.00 [0.00–2.50]; *p* = 0.023), indicating greater regression of nasal polyps. Very good disease control, defined as NPS ≤ 1, was achieved by 51.9% of patients after revision surgery and by 87.0% of patients treated with dupilumab (*p* = 0.014).

The most pronounced differences between groups were observed for quality of life, assessed using the SNOT-22 questionnaire. Patients treated with dupilumab achieved very low final SNOT-22 scores (11.96 ± 7.86), whereas patients who underwent revision sinus surgery continued to experience a substantially greater symptom burden (54.07 ± 21.68). The mean between-group difference was approximately 42 points and was both statistically and clinically significant (95% CI −51.23 to −33.00; *p* = 6.15 × 10^−11^), greatly exceeding the MCID threshold for SNOT-22.

Clinically meaningful improvement in quality of life was achieved by 85.2% of patients after revision surgery and by 100% of patients treated with dupilumab. Particularly noteworthy was the proportion of patients achieving SNOT-22 < 20, corresponding to very good symptom control and minimal impairment in quality of life [23]. This outcome was achieved by only 7.4% of patients following revision surgery, compared with 82.6% of patients treated with dupilumab.

A similar pattern was observed for olfactory function. Final Sniffin’ Sticks scores were significantly higher in the dupilumab group than in the surgical group (7.65 ± 3.11 vs. 4.78 ± 2.34; 95% CI 1.28–4.47; *p* < 0.001).

The distribution of sex was comparable between groups (44.4% vs. 47.8%; *p* = 1.00), as was the prevalence of Nonsteroidal Anti-Inflammatory Drug-Exacerbated Respiratory Disease (NERD). Asthma was numerically more common among patients treated with dupilumab (78.3% vs. 59.3%), although the difference did not reach statistical significance. Allergic rhinitis was more frequent in the surgical group (25.9% vs. 8.7%), but this difference was likewise not statistically significant.

## 4. Discussion

Since the introduction of biologic therapies for the treatment of CRSwNP, direct comparisons between revision endoscopic sinus surgery and dupilumab have remained limited. The results of our study indicate that both treatment modalities were associated with improvements in disease control, as assessed by NPS, quality of life (SNOT-22), and olfactory function. However, superior clinical outcomes were observed in patients treated with dupilumab, particularly with respect to quality of life and olfactory function.

One of the most noteworthy findings was the proportion of patients achieving a SNOT-22 score below 20, corresponding to very good symptom control and minimal impact of disease on daily functioning. This outcome was achieved by 82.6% of patients treated with dupilumab, compared with only 7.4% of patients who underwent revision sinus surgery.

The prevalence of concomitant asthma was numerically higher in the dupilumab group, which may reflect a more severe type 2 inflammatory phenotype. Given that dupilumab improves both CRSwNP and asthma-related symptoms, it cannot be excluded that this dual effect contributed, at least in part, to the observed improvement in quality of life [24,25,26,27]. As this was a retrospective study, detailed asthma-control parameters were not systematically collected and were therefore unavailable for analysis.

Olfactory function was assessed using the 12-item Sniffin’ Sticks identification test. Greater improvement was observed in patients treated with dupilumab than in those undergoing revision surgery. One possible explanation is that olfactory dysfunction in CRSwNP is related not only to mechanical obstruction of the olfactory cleft by nasal polyps but also to persistent type 2 inflammation affecting the olfactory mucosa. Biologic therapy targeting IL-4 and IL-13 may therefore address this inflammatory component more effectively than surgical treatment. Furthermore, the olfactory cleft remains a particularly challenging anatomical region for surgical management, especially in patients who have undergone multiple previous sinus surgeries [28].

The decision to initiate biologic therapy should be individualized and should take into account disease severity, previous surgical interventions, and comorbid conditions, particularly asthma and NERD. Surgery remains an important component of CRSwNP management; however, in patients with recurrent and treatment-resistant disease, biologic therapy should be considered as an alternative to repeated revision procedures [1,29].

The present study has several limitations. First, its retrospective and non-randomized design introduces the possibility of selection bias and unmeasured confounding factors. Treatment allocation was based on clinical decision-making rather than randomization. In addition, endoscopic assessments were not performed in a blinded manner, which may have introduced assessment bias. The sample size was relatively small, and all patients were recruited from a single center. Furthermore, complete baseline blood eosinophil count and total IgE data were not available for all patients, precluding their inclusion in the baseline comparison. Therefore, the findings should be interpreted with caution and require confirmation in larger prospective comparative studies.

Despite the growing body of evidence supporting dupilumab therapy, the long-term safety profile and optimal treatment duration remain to be established. In the SINUS-52 trial, the most frequently reported adverse events included injection-site reactions, arthralgia, and ocular symptoms [30]. No clinically significant adverse events related to biologic therapy were observed in our cohort. A transient increase in peripheral blood eosinophil count was observed in two patients and resolved spontaneously during follow-up.

Another interesting observation was the greater variability of outcomes following revision surgery, particularly with respect to SNOT-22 scores. This may suggest a more heterogeneous clinical response to surgical treatment compared with biologic therapy. However, this observation should be interpreted cautiously and requires confirmation in larger patient cohorts.

## 5. Conclusions

Both revision endoscopic sinus surgery and dupilumab therapy were associated with improved disease control in patients with CRSwNP. However, dupilumab treatment was associated with superior clinical disease control, greater improvement in quality of life, and better olfactory outcomes compared with revision endoscopic sinus surgery.

## Figures and Tables

**Table 1:** Baseline characteristics of the study groups.

	Revision ESS (*n* = 27)	Dupilumab (*n* = 23)	*p*
Age (years)	56.11 ± 13.10	51.83 ± 14.48	0.282
Female sex, *n* (%)	12 (44.44%)	11 (47.83%)	1.000
BMI	28.07 ± 5.95	27.64 ± 3.61	0.774
Previous ESS procedures, median (IQR)	2.00 (2.00–3.00)	4.00 (2.00–5.50)	**0.004**
Baseline NPS, median (IQR)	6.00 (5.00–6.00)	6.00 (5.00–6.00)	0.477
Baseline SNOT-22 score	75.44 ± 18.16	71.35 ± 15.49	0.394
Baseline Sniffin’ Sticks score	2.59 ± 1.05	3.00 ± 2.56	0.481
Asthma, *n* (%)	16 (59.3%)	18 (78.3%)	1.000
NERD, *n* (%)	5 (18.5%)	4 (17.4%)	1.000
Allergic rhinitis (AR), *n* (%)	7 (25.9%)	2 (8.7%)	0.152
Follow-up period (months)	14.89 ± 2.56	15.39 ± 2.44	0.482

Data are presented as mean ± standard deviation (SD), median (interquartile range, IQR), or number of patients, *n* (%), as appropriate. Bold indicates statistically significant differences (*p* < 0.05).

## Data Availability

The data are not publicly available due to privacy and ethical restrictions but are available from the corresponding author upon reasonable request.

## Abbreviations

The following abbreviations are used in this manuscript:

AR	Allergic Rhinitis
BMI	Body Mass Index
CI	Confidence Interval
CLCs	Charcot-Leyden Crystals
CRSwNP	Chronic Rhinosinusitis with Nasal Polyps
EPOS	European Position Paper on Rhinosinusitis and Nasal Polyps
ESS	Endoscopic Sinus Surgery
hpf	high-power field
IgE	Immunoglobulin E
IL	Interleukin
IL-4Rα	Interleukin-4 Receptor Alpha
ILC2	Group 2 Innate Lymphoid Cells
MCID	Minimal Clinically Important Difference
NERD	Nonsteroidal Anti-Inflammatory Drug-Exacerbated Respiratory Disease
NPS	Nasal Polyp Score
SNOT-22	Sino-Nasal Outcome Test-22
Th2	T Helper Type 2
